# Effects on deep dry needling of the TIBIALIS posterior muscle on plantar pressure distribution: A single-blinded randomized controlled baropodometric trial

**DOI:** 10.1371/journal.pone.0357010

**Published:** 2026-09-22

**Authors:** María Carmen Martínez-González, Sonia del-Río-Medina, Sergio Montero-Navarro, Jesús Sánchez-Más, María-Isabel Rocha-Ortiz, Francisco-Javier Molina-Payá, Cristina Orts-Ruiz, Cristina Salar-Andreu, Luis-Eduardo Calderón-Santos, José-Martín Botella-Rico, Jaume Morera-Balaguer

**Affiliations:** 1 Department of Medicine and Surgery Science Faculty, Universidad Cardenal Herrera-CEU, CEU Universities, Elche, Spain; 2 Physical Therapy Department, School of Health Sciences, CEU-Cardenal Herrera University, CEU Universities, Elche, Spain; 3 Department of Biomedical sciences, Health Science Faculty, Universidad Cardenal Herrera-CEU, CEU Universities, Elche, Spain; Somaiya Vidyavihar University K J Somaiya College of Engineering, INDIA

## Abstract

**Introduction:**

Myofascial trigger points (MTrPs) in the tibialis posterior muscle (TP) can create a TP dysfunction, leading to alterations in plantar pressure distribution and foot loading patterns. These alterations can be objectively evaluated through baropodometric analysis. Our hypothesis was that the application of Deep dry needling (DDN) to the TP latent trigger points (LTrPs), may reverse these changes.

**Objective:**

to investigate whether changes in plantar pressure distribution occur after the application of DDN to the TP in individuals with LTrPs, to identify the specific areas where these changes take place and to assess their persistence over time.

**Material and methods:**

A single-blind randomized controlled trial was performed. Eighty-two individuals were randomly assigned to receive either DDN (n = 48) or simulated DDN (n = 34) on the MTrPs of the TP. Participants were evaluated using dynamic baropodometry prior the procedure, immediately after, and 24 and 72 hours after.

**Results:**

Immediately after the intervention, a significant increase in rearfoot pressure was observed in the experimental group. Twenty-four hours after, this group showed an increase in mean plantar pressure, a decrease in mean pressure surface, and a decrease in forefoot contact surface. Seventy-two hours after, no significant differences with the pre-intervention data were observed for any of the variables compared.

**Conclusion:**

A single session of DDN applied to a latent myofascial trigger point in the tibialis posterior muscle produces transient changes in plantar pressure distribution, reducing foot pronation and ankle valgus. These effects peak immediately and at 24 hours but are not sustained at 72 hours. DDN may be useful as an adjunctive technique in the management of tibialis posterior dysfunction, although multi-session protocols combined with other interventions should be investigated.

ClinicalTrials.gov Identifier: NCT03756428

## Introduction

### Background

Alterations in the function of the foot’s musculoskeletal structures may lead to biomechanical imbalances and the development of future pathologies, originating from or affecting the foot itself^1^. One of the common causes of such dysfunctions is the presence of myofascial trigger points (MTrPs) [1,2]. A MTrP located in foot muscles may disrupt coordinated muscle activation, potentially altering the base of support and affecting gait mechanics^1^.

A MTrP is a hyperirritable focus located within a taut band of muscle that becomes painful when the muscle is compressed, stretched, or overstretched [3]. Their presence can cause referred pain [4,5], muscle spasm [6], decreased strength [5,7], reduced motion (ROM), and altered muscle activation patterns [5,8]. MTrPs can be classified as active trigger points (ATrPs) or latent trigger points (LTrPs), depending on the presence or absence of spontaneous pain, respectively [4, 5]. LTrPs can easily become active if the underlying causes are not treated [5]. LTrPs are frequently present in lower limb disorders [2].

The tibialis posterior muscle (TP) acts as the primary dynamic stabilizer of the medial longitudinal arch (MLA) and main inverter of the midfoot [9,10]. During the gait cycle, as soon as the foot makes contact with the ground, the tibialis posterior generates plantar flexion of the ankle, inversion and adduction of the foot, raising the MLA [11]. This function is essential for preventing hyperpronation and ensuring a solid, functional base for propulsion in the later phases of the gait cycle, contributing to movement efficiency and preventing overload on other structures of the lower limb [12].

MTrPs in this muscle can create a posterior tibial tendon dysfunction (PTTD), a foot condition characterized by loss of action of the TP muscle unit [12, 13], mainly the containment of the MLA and limitation of pronation, leading to flattening of the MLA and valgus deviation of the ankle [9,10], as well as inflammation and/or degeneration of the tendon, thus affecting walking and running and potentially leading to an acquired flat foot deformity [9,11,14–16]. PTTD is both progressive and disabling. Throughout the course of the process, it is often associated with alterations affecting the ligaments (i.e., static stabilizers). This ultimately causes the appearance or progressive increase of the deformity, which transitions from flexible to rigid. In advanced cases, with marked valgus deformity of the heel, pain may occur on the outer side of the ankle due to compromise of the lateral structures with friction between the fibular malleolus and the calcaneus [17].

This condition is among the leading causes of acquired flat feet in healthy adults and/or pain in the medial aspect of the ankle and foot, with prevalence estimates ranging between 3.3% and 10%. The actual prevalence is presumed to be higher, as early stages frequently go unrecognized or undiagnosed [12,17]. It is observed more commonly in women, in runners exhibiting excessive foot pronation, and its incidence increases progressively with age [2,14].

Decisions regarding management of this disfunction vary according to the stage of the pathology [9,13,17]. Conservative management is used in earlier stages, based on administration of non-steroidal anti-inflammatory drugs (NSAIDs), local strengthening exercises for the TP musculotendinous unit, stretching of the posterior leg muscles, and use of an orthosis to brace the foot [18]. Surgery aims to correct deformity in the later stages of the condition [19]. If the dysfunction is identified early, conservative care has shown to increase quality of life and decrease pain [20–23].

Different physiotherapy techniques exist for treatment of MTrPs, which aim to eliminate the associated pain and disability. One of the techniques used is dry needling, which involves inserting a fine needle into the muscle to reduce abnormal motor plate activity and improve muscle tissue oxygenation, thereby interrupting muscle contracture and normalizing neuromuscular activity [24–26]. High-quality studies demonstrate that dry needling can be an effective and safe method for the treatment of pain caused by MTrPs [27–29], and to improve range of motion [30]. Due to its anatomical location, the TP muscle cannot be palpated. For this reason, deep dry needling (DDN) is the most effective technique to access it.

In summary, MTrPs in the TP muscle can contribute to PTTD, leading to alterations in plantar pressure distribution and foot loading patterns, which are key factors in the onset and progression of symptoms. These alterations can be objectively evaluated through baropodometric analysis [31–35]. Therefore, our hypothesis was that the application of dry needling to TP MTrPs may reverse these changes. The aim of our study was to investigate, through baropodometric analysis, whether changes in plantar pressure distribution occur after the application of DDN to the TP muscle in individuals with LTrPs. Additionally, we sought to identify the specific areas where these changes take place and to assess their persistence over time. To our knowledge, no previous study has examined the effects of DDN on plantar pressure parameters.

## Materials and methods

### Design and participants

A single-blind randomized controlled trial was performed (registered with Clinicaltrials.gov, NCT03756428). The study was conducted in accordance with the principles of the Declaration of Helsinki on human clinical trials and was approved by the Ethics Committee from the CEU Cardenal-Herrera University (CEI13/02). The clinical trial followed the CONSORT (Consolidated Standards of Reporting Trials) extension for pragmatic clinical trials [36]. All subjects signed a written informed consent and read the general study information prior to their inclusion. Both the preliminary examination to determine the presence or absence of MTrPs in the TP and the subsequent intervention were carried out by a physical therapist trained in dry needling with over 10 years of experience in this technique.

Participants were recruited from CEU Cardenal Herrera University via emails and classroom announcements between December 1 and 11, 2018. A convenience sampling method was used. To be included, participants were required to present a latent trigger point in the TP. Exclusion criteria were as follows: (1) current or previous lower limb pathologies, deformities, or orthopedic injuries that could alter static posture or biomechanics [37,38]; (2) diagnosis of fibromyalgia, myelopathy, or radiculopathy; (3) contraindications to dry needling (e.g., coagulation disorders, uncontrollable fear of needles, or phobia of injection procedures); or (4) use of analgesic medication within 24 hours prior to participation.

The participants were randomly divided into two groups (placebo or experimental group), using simple randomization by flipping a coin [39]. Due to the nature of simple randomization by coin toss, an imbalance in group sizes occurred by chance (48 participants allocated to the experimental group and 34 to the control group). To address this imbalance, we performed supplementary analyses adjusting for age (the only baseline variable that showed a statistically significant difference between groups) using ANCOVA. Additionally, we conducted repeated-measures ANOVA (or mixed linear models when normality assumptions were not met) to examine the time × group interaction across the four assessment time points. These analyses confirmed that the main findings were robust to the observed group imbalance (Supplementary data). The study was conducted in three adjoining rooms. In one of the rooms, a first evaluator was responsible for taking pre- and post-intervention algometry measurements and enrolling participants in the study. In another room, the researcher administrating the intervention was responsible for randomizing participants and performing the DDN or simulated needling. A second evaluator was located in another room, responsible for performing pre- and post-intervention baropodometric measurements.

The necessary sample size was calculated from a pilot study with eight participants per group. This sample size for the pilot study was chosen as a pragmatic and standard approach in preliminary clinical research to estimate the effect size when no previous data exist for the intervention and primary outcome in the target population. The pilot study yielded a medium effect size (Cohen’s d ≈ 0.55) for the primary outcome (mean plantar pressure). Using G*Power software (version 3.1) [40], with α = 0.05 and statistical power = 0.80, a minimum of 34 participants per group was required. We recruited additional participants to account for potential dropouts.

To account for the repeated measures design, we additionally performed mixed linear models examining the Group × Time interaction for mean plantar pressure across the four assessment time points (pre-intervention, immediately post-intervention, 24 h, and 72 h), with age included as a covariate. These models used a random intercept for each participant to account for within-subject correlation.

#### Deep dry needling.

The location of the TP MTrPs was determined according to the criteria described by Travell and Simons [5]. The patient was placed in the supine position with slight external rotation of the lower limb, and a tape measure was used to calculate the point of union between the proximal third and the middle of a line running from the tibial tubercle to the bimalleolar line. This position is also used and recommended in MTrP manuals [5].

#### Algometry.

The Commander Algometer (JTECH Medical Industries, Midvale, UT, USA) was used to assess the pressure pain threshold in the TP, according to the protocol described by Fischer [41]. According to this protocol, to objectively determine the presence of MTrPs, the pressure pain threshold is calculated by averaging the values of three consecutive measurements taken at intervals of 20–60 seconds, and this is compared with a normal value, usually the same point on the opposite side. According to this author, the critical level of abnormality is established at a difference of 2 kg/cm² lower threshold relative to a normal control point [42].

Participants randomly assigned to the experimental group underwent DDN on the MTrPs of the TP. Once identified, the needle (AGUPUNT, CE0120, 0.30X50mm) was inserted until it passed through the MTrP, subsequently the technique known as the “fast in, fast-out technique” described by Hong was applied, in which the needle was moved up and down 4–5 mm vertical motions without rotation [43, 44] This technique incorporates the idea of speed, in which speed was applied both upon entering, to elicit local twitch responses (LTRs), and upon exiting (withdrawal of the needle back into the subcutaneous tissue, that is, outside of the muscle but not outside of the skin), to avoid the contraction and local spasm that occurs with the needle inside the taut band [5,43,45]. The fast entry and exit are repeated until the LTRs are indicating that the objective of reducing the abnormal response of the motor plate has been achieved [43].

Participants randomly assigned to the control group underwent simulated dry needling, involving pressure with the needle cannula on the identified MTrP site without skin penetration.

#### Baropodometry.

Participants were evaluated using dynamic baropodometry on four occasions: before undergoing the procedure, immediately after the procedure, and 24 and 72 hours after the procedure [46]. A modular electronic baropodometer was used (Support S.r.l. Model: Clinical MultiSensor. Place of manufacture: Rome (Italy). Year of manufacture: 2009. Serial number: 1736. Quality certificate: ISO 9001:2000 No. 12966/05/S). This device is equipped with four pressure sensors per square centimeter of platform. The walking path formed by the platform is three meters long with a central sensor surface measuring 120 centimeters long by 40 centimeters wide, which is equivalent to 19,200 pressure sensors. This electronic baropodometer has been validated for scientific research [47,48].

The protocol of the “Association Française de Posturologie” was followed [49], with control of temperature (20–23 °C), light, and sound conditions. Participants were barefoot and received standardized verbal instructions: (1) “stand at the edge of the platform upright and look straight ahead at a fixed point at eye level”; (2) “when you reach the end, stop and turn around”; (3) when the participant returned to the edge of the platform, the command “walk again” was repeated; and (4) when the participant reached the end, they were instructed to “repeat the action until I tell you to stop.” All participants were asked to walk on the platform 10 times, 5 times in one direction and 5 times in the other.

### Outcome measures

The variables defining the characteristics of the population were: sex, age, body mass index (BMI; kg/m^2^), physical activity (hours/week), and pressure pain threshold in the posterior tibial nerve prior to intervention (kg/cm^2^)

The baropodometric variables analyzed included plantar pressure and contact surface parameters: maximum plantar pressure (g/cm^2^), mean plantar pressure (g/cm^2^), mean load (%), mean contact surface (cm^2^), forefoot contact surface (cm^2^), forefoot load (%), rearfoot contact surface (cm^2^), rearfoot load (%)

The baropodometric analysis also assessed maximum pressure (g/cm^2^) and mean pressure (g/cm^2^) according to specific plantar regions: medial rearfoot (AREA A), lateral rearfoot (AREA B), lateral midfoot (AREA C), medial midfoot (AREA D), medial forefoot (AREA E), and lateral forefoot (AREA F).

#### Methods of analysis.

The mean ± standard deviation was used for the quantitative variables, and frequency tables for the qualitative data. The Kolmogorov-Smirnov test was used to determine whether the data of the variables followed a normal distribution or not. Unpaired t-test or U Mann-Whitney tests were used for two-group comparisons, as appropriate. A one-way analysis of variance or Chi-square test was used for multiple comparisons, as appropriate. To verify the homogeneity of the intervention and control groups, the association with the explanatory variables of sex, age, BMI, and hours of sports practice was measured using the Chi-square test or Student's t-test, as appropriate. All analyses were performed using SPSS Statistics version 24.0 (SPPS Inc., Chicago, IL, USA). Statistical significance was set at p < 0.05. Both per-protocol and adjusted analyses (for age) are reported.

## Results

**Fig 1:**
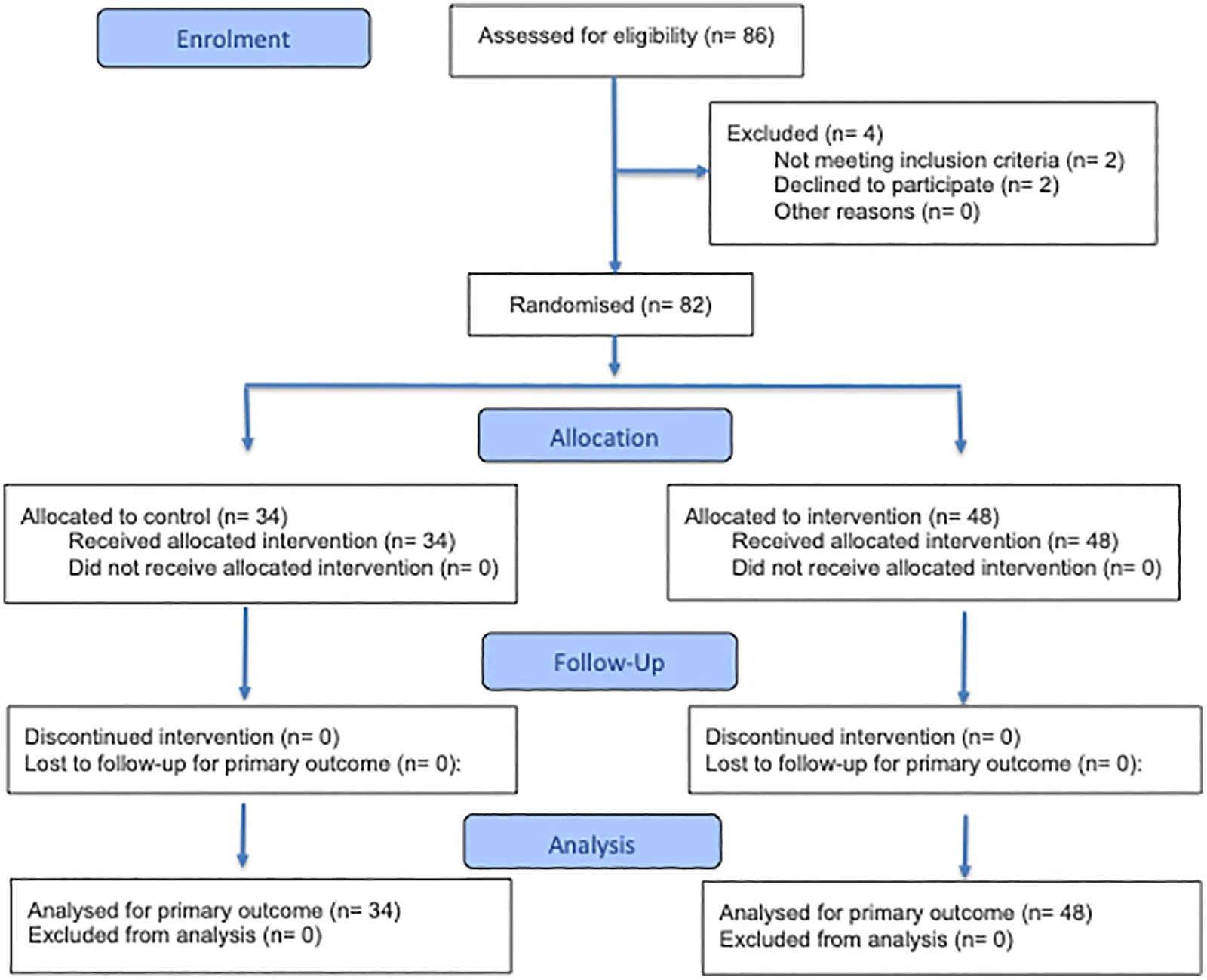
Flow diagram of the study protocol. Of the 86 individuals assessed for eligibility, 82 were randomized: 34 were allocated to the control group and 48 to the deep dry needling intervention targeting myofascial trigger points. Four participants were excluded. No participants were lost to follow-up at 24 and 72 hours post-intervention, and all randomized participants were included in the final analysis.

As shown in Fig 1, 86 subjects were screened for eligibility criteria, of which 4 subjects were excluded from the study: two failed to meet the inclusion criteria and two declined to participate (fear of needles). Finally, 82 subjects participated in the study, 34 in the control group and 48 in the intervention group (Fig 1).

Anthropometric parameters, physical activity characteristics, and algometry results for each group are shown in Table 1. Although a statistically significant difference in age was observed between groups (p = 0.039), no significant baseline differences were found in BMI, physical activity level, algometry, or baropodometric variables. To assess the potential impact of this age imbalance, we performed supplementary analyses adjusting for age (ANCOVA on key outcomes at 24 hours and mixed linear models for the Group × Time interaction). These analyses confirmed that the main within-group findings remained robust after adjustment for age (see Supplementary Data). The total population was distributed homogeneously by gender (48.8% male vs. 51.2% female), with a mean age of 22.9 ± 5.3 years, a BMI of 22.6 ± 2.5, and an average of 3.8 ± 3.9 hours/week of physical activity. A significant difference was observed between the control and experimental groups for age (p = 0.039), but not for BMI (p = 0.348) or physical activity frequency (p = 0.632).

**Table 1:** Characteristics of the studied population.

	Total (n = 82)	Control (n = 34)	Experimental (n = 48)
Population (%)	100	41.5	58.5
Sex (% men)	48.8	50.0	47.9
Age (years)	22.9 ± 5.3	24.7 ± 6.3	21.7 ± 4.2*
BMI (kg/m^2^)	22.6 ± 2.5	22.3 ± 2.2	22.9 ± 2.6
Physical activity (h/week)	3.8 ± 3.9	3.9 ± 3.5	3.8 ± 4.2
Algometry (kg)	15.0 ± 2.3	14.6 ± 2.4	15.4 ± 2.3

BMI, body mass index. Data shown as mean ± standard deviation or percentage. *p < 0.05 vs. control group.

Table 2 shows the values of plantar pressure and contact surface obtained from the baropodometric measurements at the different time points of the study. Immediately after needling of the tibialis posterior (Post) compared to baseline (Pre), a significant increase in rearfoot pressure was observed in the experimental group (p = 0.021, Post vs. Pre). Twenty-four hours after the intervention, the experimental group showed an increase in mean plantar pressure (p = 0.008, 24h Post vs. Pre), a decrease in mean pressure surface (p = 0.001, 24h Post vs. Pre), and a decrease in forefoot contact surface (p < 0.001, 24h Post vs. Pre). Longitudinal analysis using mixed linear models (adjusted for age) showed that the Group × Time interaction for mean plantar pressure was not statistically significant at any post-baseline time point (all p > 0.55). The coefficient for the Group × 24 h interaction was + 28.0 (p = 0.554). These findings indicate that the temporal trajectories did not differ significantly between groups after adjustment for age (see Supplementary Note S1 for full details in S1 Data). Seventy-two hours after the intervention, no significant differences were observed for any of the variables compared with the pre-intervention data, in either the control or experimental group.

**Table 2:** Baropodometric data on plantar pressure and contact surface parameters of the footprint.

		Pre	Post	p	24h Post	p	72h Post	p
Maximum plantar pressure (g/cm^2^)	CG	1645 ± 298	1607 ± 261	0.327	1635 ± 286	0.829	1587 ± 225	0.197
	EG	1670 ± 242	1644 ± 254	0.424	1711 ± 280	0.183	1640 ± 259	0.379
Mean plantar pressure (g/cm²),	CG	1046 ± 251	1024 ± 225	0.63	1084 ± 161	0.336	1022 ± 107	0.551
	EG	1086 ± 145	1062 ± 196	0.405	1153 ± 185	0.008	1081 ± 171	0.823
Mean load (%).	CG	50.6 ± 2.2	50.4 ± 2.3	0.485	50. ± 2.2	0.804	50.6 ± 2.4	0.875
	EG	50.3 ± 2.6	50.9 ± 2.8	0.188	50.6 ± 2.2	0.562	51.2 ± 3.5	0.13
Mean contact surface (cm^2^)	CG	64.2 ± 12.2	64.5 ± 11.6	0.426	63.1 ± 10.8	0.292	66.3 ± 12.6	0.122
	EG	70.0 ± 11.1	69.8 ± 12.6	0.758	65.6 ± 14.1	0.001	69.0 ± 15.9	0.576
Forefoot contact surface (cm^2^).	CG	33.8 ± 6.8	34.1 ± 6.6	0.565	32.9 ± 6.5	0.143	34.3 ± 6.7	0.477
	EG	36.9 ± 6.5	36.5 ± 7.2	0.443	34.9 ± 6.3	<0.001	37.2 ± 7.5	0.663
Forefoot load (%)	CG	51.0 ± 2.7	51.3 ± 2.5	0.611	50.5 ± 2.6	0.182	50.9 ± 2.6	0.761
	EG	51.4 ± 2.3	50.8 ± 2.4	0.124	50.4 ± 7.1	0.362	51.4 ± 2.7	0.954
Rearfoot contact surface (cm^2^)	CG	30.3 ± 5.8	30.9 ± 5.2	0.268	30.2 ± 4.6	0.903	31.3 ± 5.4	0.078
	EG	32.6 ± 6.5	33.3 ± 5.7	0.349	31.7 ± 5.9	0.214	32.8 ± 7.2	0.234
Rearfoot load (%)	CG	46.1 ± 2.6	46.1 ± 2.4	0.976	46.7 ± 2.4	0.154	46.6 ± 2.4	0.326
	EG	46.0 ± 2.3	46.6 ± 2.3	0.09	45.9 ± 2.7	0.947	45.3 ± 6.1	0.681

Data are presented as mean ± standard deviation. P-values correspond to intragroup comparisons versus baseline (paired t-test or Wilcoxon signed-rank test). *p < 0.05. CG, control group; EG, experimental group; Pre, before intervention; Post, immediately after intervention; 24h Post, 24 hours after intervention; 72h Post, 72 hours after intervention.

Table 3 presents the plantar pressure data differentiated by plantar footprint regions for each group and measurement time point. In the experimental group, immediately after needling of the tibialis posterior (Post) compared to baseline (Pre), a significant increase was observed in the mean pressure of regions A (p = 0.002), B (p = 0.001), C (p < 0.001), D (p = 0.001), and F (p = 0.002), along with a significant decrease in the maximum pressure of regions A (p < 0.001), B (p = 0.001), D (p = 0.016), E (p = 0.016) and F (p = 0.001). Twenty-four hours after needling (24h Post), mean pressure values in regions A (p = 0.015), B (p = 0.043) and C (p = 0.038) remained significantly higher than baseline, while maximum pressure in region D (p = 0.033) remained significantly lower. Seventy-two hours after the intervention, no significant differences were found in any of the variables compared with pre-intervention data. The control group showed no significant changes in any of the measured variables when compared with baseline values.

**Table 3:** Baropodometric data on plantar pressure by foot regions (mean ± SD).

		Pre	Post	p	24h Post	p	72h Post	p
Maximum pressure medial rearfoot (g/cm^2^) AREA A	CG	28.1 ± 4.9	28.1 ± 4.7	0.914	27.8 ± 4.5	0.554	27.7 ± 4.5	0.469
	EG	30.2 ± 5.3	27.9 ± 3.9	<0.001*	29.4 ± 5.6	0.297	29.6 ± 5.7	0.391
Mean pressure medial rearfoot (g/cm^2^) AREA A	CG	57.4 ± 8.1	58.4 ± 7.4	0.147	58.4 ± 7.5	0.145	58.3 ± 7.2	0.330
	EG	59.2 ± 8.3	62.4 ± 10.4	0.002*	62.0 ± 9.9	0.015	60.4 ± 8.0	0.188
Maximum pressure lateral rearfoot (g/cm^2^) AREA B	CG	18.0 ± 4.4	18.3 ± 4.5	0.639	17.2 ± 5.0	0.248	17.0 ± 5.9	0.213
	EG	20.4 ± 5.4	18.8 ± 5.1	0.001*	18.97 ± 5.6	0.072	19.7 ± 5.7	0.227
Mean pressure lateral rearfoot (g/cm^2^) AREA B	CG	55.6 ± 7.1	56.2 ± 5.8	0.411	56.8 ± 6.1	0.121	56.3 ± 5.6	0.382
	EG	57.8 ± 9.5	60.0 ± 9.0	0.011*	59.9 ± 8.2	0.043	58.5 ± 7.7	0.43
Maximum pressure lateral midfoot (g/cm^2^) AREA C	CG	27.3 ± 4.5	27.0 ± 4.8	0.591	26.5 ± 5.3	0.211	27.2 ± 5.1	0.859
	EG	28.6 ± 4.8	28.5 ± 5.9	0.858	28.3 ± 5.3	0.616	29.3 ± 5.3	0.173
Mean pressure lateral midfoot (g/cm^2^) AREA C	CG	58.9 ± 7.6	59.9 ± 7.0	0.079	60.4 ± 7.2	0.123	60.5 ± 7.2	0.110
	EG	60.1 ± 8.8	63.5 ± 10.4	<0.001*	62.7 ± 10.1	0.038	61.1 ± 7.5	0.235
Maximum pressure medial midfoot (g/cm^2^) AREA D	CG	21.8 ± 4.9	22.7 ± 4.5	0.138	21.5 ± 4.0	0.498	21.7 ± 5.5	0.789
	EG	24.0 ± 5.5	22.9 ± 5.2	0.016*	22.9 ± 4.3	0.033	23.45 ± 4.3	0.220
Mean pressure medial midfoot (g/cm^2^) AREA D	CG	59.3 ± 7.9	59.7 ± 7.5	0.55	61.0 ± 8.6	0.057	60.6 ± 7.82	0.179
	EG	61.3 ± 9.8	64.3 ± 11.4	0.001*	63.0 ± 11.2	0.188	61.6 ± 8.0	0.748
Maximum pressure medial forefoot (g/cm^2^) AREA E	CG	5.5 ± 7.9	3.3 ± 2.7	0.17	3.4 ± 2.8	0.177	3.0 ± 2.1	0.071
	EG	4.7 ± 3.5	3.2 ± 2.7	0.016*	4.0 ± 3.4	0.738	4.4 ± 4.1	0.912
Mean pressure medial forefoot (g/cm^2^) AREA E	CG	55.26 ± 7.2	54.5 ± 5.0	0.745	55.8 ± 5.6	0.901	52.7 ± 10.9	0.311
	EG	54.33 ± 11.5	54.0 ± 11.5	0.798	54.6 ± 14.7	0.889	56.7 ± 7.2	0.119
Maximal pressure lateral forefoot (g/cm^2^) AREA F	CG	42.0 ± 6.3	41.8 ± 6.0	0.706	41.4 ± 5.9	0.363	40.6 ± 7.4	0.082
	EG	44.5 ± 7.0	42.4 ± 8.0	0.001*	44.0 ± 7.8	0.495	44.9 ± 8.1	0.543
Mean pressure lateral forefoot (g/cm^2^) AREA F	CG	59.5 ± 7.1	60.14 ± 6.4	0.393	60.6 ± 6.7	0.291	60.9 ± 7.2	0.224
	EG	59.9 ± 12.1	64.0 ± 10.5	0.002*	63.7 ± 13.6	0.101	62.7 ± 8.0	0.055

Data are presented as mean ± standard deviation. P-values correspond to intragroup comparisons versus baseline (paired t-test or Wilcoxon signed-rank test). *p < 0.05.

CG, control group; EG, intervention group; Pre, before intervention; Post, immediately after intervention; 24h Post, 24 hours after intervention; 72h Post, 72 hours after intervention.

## Discussion

The current randomized controlled trial demonstrated that the application of a single session of DDN induced changes in plantar pressure distribution during gait by reducing pronation and valgus of the foot in patients with LTrPs in the TP muscle. Previous studies have demonstrated changes in plantar pressure distribution following dry needling. Martínez-Jiménez et al [50] demonstrated that after dry needling of the flexor digitorum brevis muscle, the mean pressure in the midfoot increased, the surface area in the forefoot increased, and the maximum pressure in the rearfoot decreased. This study only measured the plantar pressure distribution before and immediately after the intervention, therefore, it is unknown whether its results at 24 and 72 hours would have been comparable to ours. The transient nature of the observed effects (significant up to 24 hours but returning to baseline at 72 hours) suggests that a single session of DDN may act as a useful “priming” intervention. Combining DDN with strengthening exercises of the tibialis posterior, foot orthoses, or taping could help maintain the biomechanical improvements. Future studies should evaluate multi-session protocols and longer follow-up periods to determine clinical relevance in patients with symptomatic posterior tibial tendon dysfunction.

However, these changes were not maintained at the 24-hour or 72-hour follow-up assessments. We believe that this finding may be due to several factors:

1) The study participants did not present any previous deformities or symptoms that could be attributed to MTrPs in the TP, as this was an inclusion criterion. Therefore, the change could not be maintained, as it may not have produced an improvement in function.
2) The MLA is maintained by the action of various ligaments and muscles (tibialis posterior, peroneus longus, flexor digitorum longus, flexor hallucis longus, and flexor digitorum brevis muscles) [51,52], many of which have not been addressed.
3) No intervention addressed the compensatory activity of other muscles (peroneals, tibialis anterior, and gastrocnemius) commonly associated with DTTP [53].
4) A single needling session applied to one MTrP of the TP muscle may have been insufficient to induce sustained effects.
5) Between the post-intervention and 24-hour assessments, our patients resumed wearing their own footwear, which was often adapted to their individual foot mechanics. Re-exposure to these worn-in shoes may have promoted a return to their habitual gait patterns [54].
6) We know that each individual has a habitual gait pattern, influenced by their neuromuscular system, biomechanics, and habits acquired over time. This tendency is attributed to motor memory and neuromuscular efficiency, which favor the repetition of previously learned and automated movements [55–57]. However, in our study we did not carry out any treatment aimed at changing this gait pattern.
7) The individuals in the sample spent a significant average number of hours per week doing sport (3.8 in the intervention group). Perry et al. [58] compared the myoelectric activity of the TP muscle during slow walking, walking at a free pace, and fast walking. The results showed that EMG activity increased directly with increasing walking speed. Baropodometric measurement is performed with normal walking, however, it is possible that in the 24 hours between the post measurement and the subsequent measurement, many of the individuals in our sample may have engaged in sports activities involving fast walking, thereby increasing the muscle tone of this muscle by increasing EMG activity.

None of the above detracts from our findings, as we demonstrate that a very simple, rapid, and inexpensive procedure can improve plantar pressure distribution by increasing the height of the MLA, although other measures may need to be added to maintain this improvement. Changes in the height of the foot’s MLA are associated with a greater prevalence of lower limb injuries [59], including plantar fasciitis [60, 61], knee flexor injuries [60], triceps surae injuries [60], noncontact anterior cruciate ligament injuries [62], lateral ankle sprains [63], foot pain [64] and bony injuries [65], which demonstrates the importance of investigating any approach that could improve this factor. Finally, PTTD is considered the most common cause of adult-acquired flatfoot [66,67].

In our study, we observed a high prevalence of MTrPs in the TP muscle. These findings are consistent with those reported by other researchers in the field. Zuil-Escobar JC et al. [2] identified a 77.7% prevalence of of MTrP in lower limb muscles among asymptomatic subjects. Although their study used a different detection protocol [5], the most frequent diagnostic criteria were the presence of a taut band and a tender spot, reported in 98–100% of cases. In another study, the same authors analyzed the prevalence of MTrPs in several muscles of the lower limb in subjects with a lower MLA, finding that 73% of subjects presented MTrPs in the lower limb. Moreover, they reported that a lower MLA was associated with a higher prevalence of MTrPs in the lower limb.

A strength of our study is that, to our knowledge, it is the first to analyze the influence of MTrPs in the TP on plantar pressure and footprint characteristics. This is probably related to the challenges involved in treating the TP due to its deep anatomical location. Our findings demonstrate that the effects of dry needling on the TP can be objectively assessed through baropodometric analysis. Therefore, this study provides a foundation for further research in this field.

### Study limitations

Our study had several limitations. Although a chance imbalance in age was observed between groups, supplementary analyses adjusting for this variable demonstrated that the primary findings were not materially influenced by this difference. Additional limitations include the use of a convenience sample of young, mostly asymptomatic university students, which limits generalizability to clinical populations with established foot pathology. Further studies in populations with different demographic characteristics or foot pathologies are needed to extrapolate the results of this research.

The fact that the TP is a very deep muscle limited the detection of its trigger points. Nonetheless, the person in charge of their detection was sufficiently trained for this, minimizing this limitation.

It would be interesting for future research to investigate the effect on plantar pressure distribution in patients with MTrPs in TP using a treatment protocol that considers not only this muscle but also those associated with maintaining the height of the MLA. Similarly, future studies could employ protocols involving more than one session of DDN applied to the MTrPs of the TP. Finally, the relatively short follow-up period (72 hours) does not allow conclusions regarding longer-term effects; it would be relevant to conduct subsequent studies to determine the effectiveness of the intervention in the medium and/or long term.

## Conclusions

A single session of DDN applied to a MTrP in the TP muscle produces measurable changes in plantar pressure distribution, reducing foot pronation and ankle valgus. Although these effects do not persist beyond 24 hours, the results suggest that DDN could be a useful therapeutic strategy for the acute management of posterior tibial dysfunction and to prevent other lower limb injuries, warranting further investigation into its long-term application.

## Supporting information

S1 File Study Plan Protocol Tibialis posterior ECA SIN LOGO. (PDF)

S1 Data Supplementary data. (DOCX)

S1 Checklist CONSORT 2025 checklist. (PDF)
