# Supplementary material for: Effects on deep dry needling of the TIBIALIS posterior muscle on plantar pressure distribution: A single-blinded randomized controlled baropodometric trial
Source: PLoS One. 2026 Sep 22;21(9):e0357010. doi: 10.1371/journal.pone.0357010 (PMC13596803; doi:10.1371/journal.pone.0357010)
Supplement: Supplementary file 1 [file pone.0357010.s001.pdf]

STUDY PROTOCOL PLAN AND STADISTICAL ANALYSIS PLAN (SAP)

UNIVERSITY CEU CARDENAL HERRERA

Principal investigator: Dr. Sergio Montero Navarro

TITLE: Effects on Plantar Support After Deep Dry Needling in Posterior Tibial Muscle: Baropodometric study

Research and Ethics Committee of CEU Cardenal Herrera University Number: CEI13/02

NCT ID: [not yet assigned]

DATE: 3-18-2013

STUDY PROTOCOL PLAN

Objectives:

The objectives of our study have been the following:

General:

- Evidence changes in pressure in the plantar support in dynamics after treatment with deep dry needling in the tibialis posterior myofascial trigger point.

Specific:

- Analyze the variation of the plantar dynamics in the subjects without intervention to avoid learning bias of the methodology of the baropodometric registry.
- Evidence if deep dry needling is effective in the treatment of myofascial trigger point.
- Observe if there are differences in the treatment taking into account the covariates measured such as age, sex, and physical activity practice and body mass index.

Volunteers who want to participate in the study will read the general information of the study and sign the informed consent to participate in the study.

Once the informed consent has been read and delivered, the evaluator will check that they meet the inclusion criteria. After the data collection, a randomization (Epidat V4.0) of the selected subjects will be carried out to assign them to one of the intervention groups.

In the evaluation room, the evaluator, after taking the measurements of weight and height.

To locate the myfascial trigger point in the tibialis psoterior, the palpation technique will be used, grasping the belly of the muscle between the thumb and the 2nd and 3rd fingers. The evaluator will press the fibers with a forward and backward movement to find the taut bands. Once the taut band is found longitudinally, the nodule and the point of greatest sensitivity to pressure will be located.

Once the myofascial trigger point is located, it will be marked with a dermal marker. Those subjects in whom the myofascial trigger point can not be located with precision, will be excluded from the study.

After having marked the myofascial trigger point, the evaluator will make the subject the algometry in the myofascial trigger point. 3 measurements will be taken with an interval of 20 seconds between measurement and measurement, and the average of the three measurements obtained will be calculated.

Next, The baropodometry will be carried out. The subject will perform the march on the platform for one minute. At the moment that the baropodometer registers correctly the data will warn us.

Once the pre-intervention measurements have been made, the subject will go to the adjoining room to carry out the intervention.

The intervention sequence varied depending on the group to which the subject "Group intervention 1" (G1),

- "Intervention group 1" (G1): the deep dry needling technique will be performed with a duration of less than one minute in the tibialis posterior.

Initial position: The patient will be placed supine with an external rotation of the hip and slight knee flexion. The auditor is placed on the homolateral side.

Placement of hands: the auditor locates the myofascial trigger point in the tibialis posterior with the left hand on the inside, back of the tibia, and perform the technique with the right hand.

Technique: The inspector, with gloves, disinfects the area to be treated with 70º alcohol. Once the alcohol is applied, the intervenor locates and takes the myofascial trigger point and places the needle with the guide tube resting on the skin of the patient. The inspector holds the guide tube between the index and middle fingers lightly touch the needle with the index finger of the other hand to insert the needle perpendicularly into the skin, directing it towards the inspector's thumb. Once the needle is inserted subcutaneously, the guide tube is removed. The controller deepens the needle to myofascial trigger point, and makes fast inputs and outputs into it. The rapid exit is made to the subcutaneous cellular tissue, outside the muscle, but not outside the skin. Next, hemostasis is performed in the area. Once this hemostasis is completed, the patient is incorporated and dressed.

- "Control group (G2)": the simulated dry puncture technique will be performed.

Initial position: The patient will be placed prone. The interventor is placed on the homolateral side.

Technique: The patient lies on the stretcher without the trousers. The interventor, with the guide tube, simulates the technique of puncture in the right twin of the subject. The comptroller cleans the area with 70º alcohol. Once the alcohol is applied, he performs the simulated dry needling technique in a plane with his index and middle fingers with the guide tube. It is important that the subject has his head stuck in the hollow of the head of the stretcher throughout the technique to make a correct masking.

Next, a simulation of hemostasis is performed in the area. Once this simulation is finished, the patient is incorporated and dressed.

Once the intervention is done, the subject will return to the evaluation room to proceed to the evaluation immediately after the intervention (POSTINT), both algometric and baropodometric measurements. After these measurements, the subject will be summoned at the same time to return at 24 hours (POST24h) and at 72 hours (POST72h) to perform the corresponding algometric and goniometric measurements.

This study will be carried out in two adjoining rooms, with a sufficient amplitude to offer freedom of movement, both to the evaluator and the auditor and the subject of the study.

In one of the rooms will be the evaluator to perform the measurements and collect the pre- and post-intervention values. In the adjoining room will be the controller, in charge of carrying out the corresponding intervention.

Between both rooms there are about eight meters of distance without steps or unevenness, and they will maintain an identical and constant temperature.

Statistical Analysis Plan (SAP)

For the qualitative variables we will make some frequency tables. On the other hand, for the quantitative variables, maximums, minimums, mean values and the standard deviation will be calculated.

To check the homogeneity of the intervention and control groups, the association with the explanatory variables sex, age, body mass index and sport hours will be measured using the Chi-square test or the T-student test as appropriate.

The equality of mean values of each dependent variable will be tested, both in the intervention group and in the control group, as in the four measurement times by means of an ANCOVA procedure (analysis of the covariance with repeated measures).

The confidence interval will be 95%

GENERAL STUDY INFORMATION AND INFORMED CONSENT

UNIVERSITY CEU CARDENAL HERRERA

Principal investigator: Dr. Sergio Montero Navarro

TITLE: Effects on Plantar Support After Deep Dry Needling in Posterior Tibial Muscle: Baropodometric study

Research and Ethics Committee of CEU Cardenal Herrera University Number: CEI13/02

NCT ID: [not yet assigned]

DATE: 3-18-2013

GENERAL STUDY INFORMATION

TITLE: **Effects on Plantar Support After Deep Dry Needling in Posterior Tibial Muscle: Baropodometric study**

Mr. Sergio Montero Navarro, Physiotherapist, principal investigator and researcher reports that:

The study in which will be part of joint mobility and pain at the foot. In this way we can contribute to promote the health status of people. A response inmediately after intervention, at 24 hours and 72 hours after the intervention.

The tests performed are simple and in no case involve difficulty, fatigue, danger, injury, pain or adverse reaction. They will be held in the practice room of the CEU Cardenal Herrera University in Elche, specially prepared for the occasion and in the best conditions of safety and hygiene and always using approved material.

They will be carried out by collegiate physiotherapists in the School of Physiotherapists of the Valencian Community.

The general data of the subject will be collected (name, age, sex, physical variables and clinical history). The article must be sent with comfortable clothes The day that sea cited by the researcher, previous notice. Personal data is recognized in this study.

The personal data are confidential, apply to the protection of personal data (Organic Law 15/1999, December 13) and any other thing that may be applicable.

This study was approved by the Research and Ethics Committee of CEU Cardenal Herrera University.

INFORMED CONSENT

Mr/Mrs.. with Number identification .... freely and voluntarily, I DECLARE:

That I have read the information contained in this document about the general information of the study.

I have been informed that all tests are simple to perform and do not produce harmful effects on health. They will be carried out in appropriate facilities and will be carried out by qualified and specialized personnel.

I have also been informed that, the data collected in this study will be treated confidentially, applying the current legislation on protection of personal data (Organic Law 15/1999, of December 13) and any other applicable.

Therefore, I give my consent and I authorize Mr. Sergio Montero Navarro, to carry out the detailed study in this document with the help of the necessary personnel with the appropriate qualification and specialization.

In Elche, to ...... of .... of 201

Firm
