# Supplementary material for: Effects on deep dry needling of the TIBIALIS posterior muscle on plantar pressure distribution: A single-blinded randomized controlled baropodometric trial
Source: PLoS One. 2026 Sep 22;21(9):e0357010. doi: 10.1371/journal.pone.0357010 (PMC13596803; doi:10.1371/journal.pone.0357010)
Supplement: Supplementary file 3 [file pone.0357010.s002.docx]

**Supplementary Data**

**Supplementary Table S1. Analysis of covariance (ANCOVA) for baropodometric parameters at 24 hours post-intervention, adjusted for age and baseline values.**

| **Parameter** | **Adjusted mean difference (Experimental minus Control)** | **95% CI** | **p-value** |
| --- | --- | --- | --- |
| Mean plantar pressure (g/cm²) | 46.42 | −27.97 to 120.80 | 0.218 |
| Forefoot contact surface (cm²) | −0.32 | −1.92 to 1.28 | 0.690 |
| Mean contact surface (cm²) | −2.32 | −5.92 to 1.28 | 0.204 |

**Note:** ANCOVA models included group allocation, age (centered at the sample mean), and the corresponding pre-intervention value as covariates. Positive values indicate higher values in the experimental (deep dry needling) group compared with the control group. CI = confidence interval.

*ANCOVA = analysis of covariance.*

After adjusting for age and baseline values, no statistically significant differences were observed between groups at 24 hours. This confirms that the main findings of the study (within-group changes in the experimental group) were not biased by the age imbalance.

**Supplementary Note S1. Mixed linear model results for mean plantar pressure (Group × Time interaction, adjusted for age).**

A mixed linear model was fitted to examine the Group × Time interaction for mean plantar pressure across the four assessment time points (pre, post, 24 h, and 72 h), with age included as a covariate. The Group × Time interaction terms at all three post-baseline time points were non-significant (all p > 0.55). Specifically, the coefficient for the Group × 24 h interaction was +28.0 (p = 0.554). The non-significant Group × Time interaction indicates that the temporal trajectories of mean plantar pressure did not differ meaningfully between the experimental and control groups after adjustment for age.
